# Development of Multifunctional Nanoparticles for Therapy and/or Diagnosis

**DOI:** 10.3390/nano12142321

**Published:** 2022-07-06

**Authors:** Sandrine Cammas-Marion

**Affiliations:** 1 Univ Rennes, Ecole Nationale Supérieure de Chimie de Rennes, CNRS, ISCR, UMR 6226, ScanMAT, UMS2001, 35000 Rennes, France; sandrine.marion.1@ensc-rennes.fr; 2 INSERM, INRAE, Univ Rennes, Institut NUMECAN (Nutrition Metabolisms and Cancer) UMR_A 1341, UMR_S 1241, 35000 Rennes, France

The design of multifunctional nanoparticles for diagnostic and/or therapeutic purposes continues to be a subject of tremendous research. Indeed, such nanocarriers associate the unique properties of multifunctional nanoparticles, which can be specifically designed for the site-specific delivery of various molecules, to those of diagnostic and/or therapeutic drugs.

This Special Issue aims to provide some recent advances in the development of those multifunctional nanovectors for diagnostic and/or therapeutic applications.

In this Special Issue, there are research articles focusing on the preparation and characterization of functional nanovectors for site-specific anti-cancer drug delivery [1,2,3] and review articles on the uses of polymeric nanoplatforms for the targeted delivery of imaging and therapeutic molecules [4] and biomedical applications [5] and on recent advances in the design of phthalocyanine loaded polymeric nanoparticles for cancer photodynamic therapy [6].

We think that the results presented in this Special Issue might be useful for researchers working in the field of nanoparticles design for diagnostic and/or therapeutic purposes.

Lastly, I would like to sincerely thank all the authors who contributed to the success of this Special Issue with respect to the quality of their research and manuscript.
